# Uniportal Video-Assisted Left Upper Segmentectomy With a Minimally Invasive Chest Wall Resection Technique for Pancoast Lung Cancer

**DOI:** 10.1016/j.atssr.2025.06.018

**Published:** 2025-07-22

**Authors:** Fumiaki Watanabe, Teruhisa Kawaguchi, Yasuhisa Urata, Iwao Hioki, Katsutoshi Adachi, Tomoaki Sato

**Affiliations:** 1 Department of Thoracic Surgery, Mie Chuo Medical Center, Tsu, Japan

## Abstract

Owing to the proximity of vital organs, apical lung cancer requires a surgical approach distinct from that used for conventional radical lung cancer surgery. Various techniques have been developed to address this challenge. The open chest approach involves extensive muscle dissection, often leading to an unexpected degree of respiratory function loss. This report outlines a surgical technique performed on a patient with reduced pulmonary function who underwent left upper division segmentectomy combined with resection of the first and third ribs. The procedure was conducted by a combination of uniportal video-assisted thoracic surgery and a localized high posterolateral incision.

Various surgical approaches have been developed for apical lung cancers to avoid intraoperative injury to vital organs and to minimize muscle dissection, which may result in postoperative respiratory function loss. This paper describes a minimally invasive uniportal video-assisted thoracic surgery (U-VATS) technique for a Pancoast tumor.

## Technique

**Figure 1:**
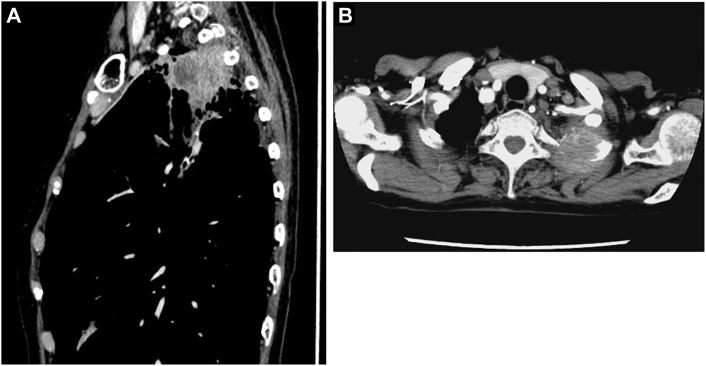
(A) Sagittal computed tomography image depicting a necrotic tumor extending to the apex of the thoracic cavity. (B) Contrast-enhanced computed tomography image showing a 56-mm tumor invading the second rib.

A 65-year-old woman presented to our hospital with complaints of bloody sputum, left thoracic back pain, and an abnormal sensation in the left upper limb. Computed tomography imaging of the chest revealed a substantial 56-mm lesion with necrosis at the apex of the left lung, with invasion into the first intercostal space (Figure 1A) and the second rib (Figure 1B; Video 1). A bronchoscopic biopsy confirmed a diagnosis of non-small cell lung cancer. Induction chemoradiotherapy was administered, consisting of 3 courses of carboplatin (area under the free carboplatin plasma concentration vs time curve = 2) and paclitaxel (40 mg/m^2^), along with radiation therapy (40 Gy/20 fractions) targeting the primary lesion at the lung apex. On post-neoadjuvant restaging imaging, the main lesion had developed a cavity containing a structure suggestive of necrosis. In addition, there was a reduction in wall thickening at the border between the tumor and the chest wall (Video 2). After treatment, the forced expiratory volume in 1 second (FEV_1_) was 1230 mL, with a percentage predicted FEV_1_ of 40.2%. The patient’s diffusing capacity of the lung for carbon monoxide (Dlco) was also reduced, measuring 7.68 L, with a percentage predicted Dlco of 58.4%. The predicted postoperative FEV_1_ was 971 mL for left lobectomy and 1100 mL for left upper division segmentectomy. Similarly, the predicted postoperative Dlco was 46.1% for lobectomy and 52.3% for segmentectomy.

**Figure 2:**
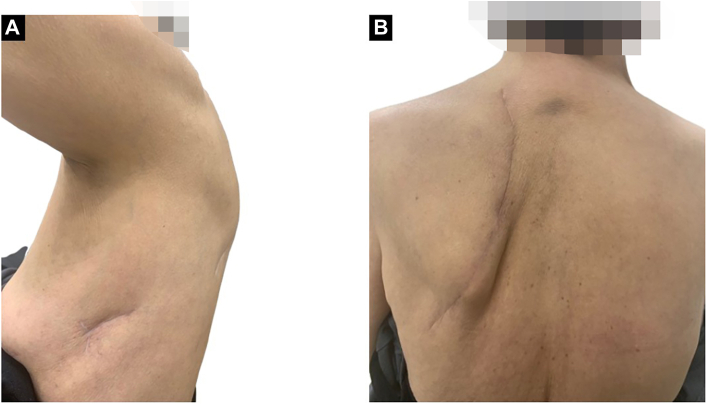
(A) A 3-cm incision was made along the midaxillary line at the fifth intercostal space. (B) A skin incision was created from the shoulder opening to immediately posterior to the inferior angle of the scapula, following the line of the posterolateral incision.

Considering the patient’s age and coexisting pulmonary emphysema, left upper division segmentectomy was selected as the preferred surgical approach. A uniport was inserted into the fifth intercostal space (Figure 2A; Video 3, 00:00), and a localized high lateral incision was created (Figure 2B; Video 3, 02:06).

The S1+2c/S6 interlobar space was dissected with an ECHELON FLEX Powered Stapler (Ethicon). V1-3 was identified on the ventral side of the mediastinum and divided with a stapler, employing an ECHELON FLEX 7 Powered Stapler (Ethicon). The branches of A3 were identified and divided with the stapler. An A1+2 transection was performed to facilitate the release of the upper lobe because it could not be unfixed otherwise. The bone was dissected sequentially from the third rib to the first rib. The intercostal muscles, nerves, and arteriovenous veins were dissected with an ENSEAL X1 Tissue Sealer (Ethicon). The dorsal ribs (1-3) were dissected with a rib cusp knife, whereas the ventral ribs were dissected with a drill (Video 3, 02:36). The pulmonary apex was carefully separated from each rib, with particular attention to its dissection from the brachiocephalic artery (Video 3, 03:35). The A1+2 and upper major bronchi were dissected, and the intersegmental areas were identified with a near-infrared fluorescence endoscope (ENDOEYE [Olympus Corporation]; Video 3, 06:50). The left upper division segment was resected and removed (Video 3, 08:10). The resected chest wall was examined thoracoscopically (Video 3, 08:20). The operation lasted 6 hours 42 minutes, and the total blood loss was 734 mL. Pathologic evaluation confirmed that the tumor and dissected margins were sufficient, as chemoradiotherapy had eliminated viable cells in the vicinity. The shortest pathologic distance from the tumor to the dissected edge was at least 2 cm, and the only area that appeared indistinct from tumor on computed tomography imaging was a fibrotic scar without viable cells.

The thoracic drain was removed on postoperative day 8. However, an intrathoracic infection subsequently developed; thoracic drain reinsertion was required on postoperative day 12. *Staphylococcus* was identified in the drain fluid, and intrapleural lavage was performed. The drain was removed on postoperative day 29, and the patient was discharged in an ambulatory state on postoperative day 31.

## Comment

After the initial report by Pancoast^1^ in 1924, lung cancer invading the apex of the chest wall was historically regarded as an incurable disease with a poor prognosis. However, in the 1960s, it was demonstrated that the disease could be effectively controlled locally and cured through radiotherapy followed by surgical resection. Combined treatment with radiotherapy and surgery subsequently became the standard approach for Pancoast tumors.^2^^,^^3^ When the lesion extends from the deep subclavian region to the superior mediastinum and requires revascularization of the subclavian vessels or extensive dissection, an anterior approach is used. Conversely, if the lesion is located dorsally, a high posterolateral approach or posterior transaxillary (hook) approach is typically preferred.^4^ The posterior approach is frequently used because most Pancoast tumors are located in the midline to posterior region. In the hook approach, the subclavian artery is clearly visualized, allowing resection and reconstruction on either side without difficulty. In contrast, the posterior approach enables observation of the subclavian artery from the dorsal side but does not provide an anterior view, making direct observation challenging. Therefore, depiction of the lungs within the thoracic cavity by U-VATS combined with a specular imaging is advantageous.^5^ The posterior approach often involves extensive muscle and chest wall dissection as well as intercostal opening, which can impair respiratory function. In the method described here, dissection on the dorsal side is limited to the rhomboid and vastus lateralis muscles.^6^ Furthermore, skin incisions are minimal, even with the inclusion of radiation therapy.

In conclusion, this report demonstrates that the incorporation of a U-VATS technique facilitates minimal muscle incision during Pancoast tumor surgery. Furthermore, the thoracoscopic operative field can be directly visualized by surgeons, enhancing surgical safety.
